# Gait Analysis of Patients Subjected to the Atrophic Mandible Augmentation with Iliac Bone Graft

**DOI:** 10.1155/2019/8203597

**Published:** 2019-03-03

**Authors:** Erol Cansiz, Derya Karabulut, Suzan Cansel Dogru, Nazif Ekin Akalan, Yener Temelli, Yunus Ziya Arslan

**Affiliations:** ^1^Faculty of Dentistry, Department of Oral and Maxillofacial Surgery, Istanbul University, Istanbul, Turkey; ^2^Faculty of Engineering, Department of Mechanical Engineering, Istanbul University-Cerrahpasa, Istanbul, Turkey; ^3^Faculty of Health Science, Physiotherapy and Rehabilitation Division, Istanbul Kultur University, Istanbul, Turkey; ^4^Istanbul Faculty of Medicine, Department of Orthopedics and Traumatology, Istanbul University, Istanbul, Turkey

## Abstract

In this study, we aimed to quantitatively monitor and describe the gait functions of patients, who underwent iliac crest bone grafting in atrophic jaw augmentation operation, by taking into account the alterations of gait parameters and muscle forces in the early recovery course. To do so, temporospatial and kinematic gait parameters of ten patients during pre- and postoperative periods were recorded, and forces of the gluteus medius, gluteus maximus, and iliacus muscles were calculated. Three postoperative periods were specified as one week (post-op1), two weeks (post-op2), and three weeks (post-op3) after the surgery. Restoring process of the gait patterns was comparatively evaluated by analyzing the gait parameters and muscle forces for pre- and postoperative periods. Temporospatial and kinematic parameters of post-op3 were closer to those obtained in pre-op than those in post-op1 and post-op2 (*p* < 0.05). Muscle forces calculated in post-op3 showed the best agreement with those in pre-op among the postoperative periods in terms of both magnitude and correlation (*p* < 0.05). In conclusion, the patients began to regain their preoperative gait characteristics from the second week after surgery, but complete recovery in gait was observed three weeks after the surgery.

## 1. Introduction

Bone grafting or bone harvesting is a procedure to augment deficient bone tissue and is widely used in a number of oral and maxillofacial procedures such as reconstructive surgical interventions [1–4]. Functional and structurally sound bone volume is essential for the reconstruction of alveolar defects. For this reason, the iliac crest, calvarium, tibia, fibula, and ribs have been used as extraoral donor sites for bone augmentation procedures in the field of oral and maxillofacial surgery [5, 6]. However, the iliac crest is regarded as a gold standard on this specific field among other free bone graft donor sites [7–9]. Due to its high bone volume, relative ease of operation, and low morbidity and complication prevalence, the iliac crest is the most preferred and well-known donor site in such operations [10, 11].

Although the anterior iliac crest bone is a convenient donor site for the atrophic mandible augmentation technique, various complications associated with the donor site like chronic pain, contour defect, ureteral injury, sensory loss, and unbalance of the sacroiliac joint have been reported [5, 6, 12, 13]. Due to the operation-caused trauma that occurred in neighboring anatomical structures especially in muscle-bone connection sites, gait abnormalities are observed in the postoperative periods [14].

Restoration of severe atrophic mandible for the rehabilitation of patients with dental implant-aided fixed prosthodontics must be three-dimensional. The high-volume donor site is required for the restoration of the atrophic mandible. The reconstruction of severe alveolar defects or hypertrophic jaw bones mostly requires tricortical structure of 6 × 5 cm average graft size for augmentation [15, 16]. At this point, the gluteus medius (GMED), gluteus maximus (GMAX), and iliacus (ILIAC) muscles must be individually separated from the muscle-bone connection surface to harvest sufficient bone graft, which leads to the gait abnormalities. Since there are many factors affecting the recovery process, it is difficult to identify the healing process exactly.

Many researchers reported the gait disturbance caused by iliac crest bone grafting operations. Matsa et al. [17] reported that 28% of their study group suffered from gait disturbance in the first four weeks following surgery, and all of the patients returned to their normal gait characteristics after three months of the healing period. On the other hand, Beirne et al. [18] and Sudhakar et al. [19] reported that most of their patients who had undergone iliac crest bone grafting regained their preoperative gait characteristics within two weeks following surgery. Beirne et al. [18] and Sudhakar et al. [19] stated that only 6% and 2% of the patients suffered from gait disturbance more than two weeks after bone grafting operation, respectively. Rawashdeh [20] reported that none of their patients had gait disturbance after two weeks. None of the studies in the literature investigated the postoperative gait deficiency from objective and quantitative perspectives, and no consensus has been reached on the gait pattern in the early recovery period following the iliac crest bone harvesting.

In this respect, we aimed to quantitatively evaluate the gait functions of the patients, who underwent atrophic jaw augmentation operation in which the iliac crest was used as donor site for the augmentation procedure, in the early recovery period. Our purpose was also to describe the biomechanical alterations of the patients by taking into account temporospatial and kinematic gait parameters as well as forces of the GMED, GMAX, and ILIAC muscles. We hypothesized that the changes in the gait patterns and muscle forces would quantify the progress of the gait recovery process.

## 2. Materials and Methods

### 2.1. Patients

This prospective study included ten systemically healthy adult patients (five males and five females, aged 43 ± 10.4 years old, height 169 ± 10 cm, mass 71.2 ± 19.6 kg), who underwent onlay free bone grafting with anterior iliac crest for the rehabilitation of severe maxillary alveolar atrophy between April 2016 and April 2017 at the Istanbul University Faculty of Dentistry, Department of Oral and Maxillofacial Surgery, Istanbul, Turkey.

The inclusion criteria for the patients were as follows:

- Requirement to anterior iliac crest bone grafting for maxillary alveolar bone reconstruction due to severe alveolar bone atrophy
- Having no tumor or trauma in the lower limbs or ilium
- Having no any other neural or muscular disorder which may merge with gait disturbance
- Having no cognitive deficiency which may prevent understanding and performing of the study protocol

The exclusion criterion was the presence of any systematic disease that may affect the soft and hard tissue healing. The study protocol followed the Declaration of Helsinki and was approved by the ethical committee of Istanbul University, Istanbul, Turkey (approval protocol no. 2016/7). Written informed consent was obtained from all the patients.

### 2.2. Surgical Intervention

All of the patients were operated at the Istanbul University, Faculty of Dentistry, Department of Oral and Maxillofacial Surgery. A standardized surgical protocol to harvest approximately 6 cm × 3 cm × 2 cm tricortical free bone graft from the right anterior iliac crest was performed by the same surgeon under general anesthesia. The skin incision and dissection were performed 2 cm above the anterior superior iliac spine along the anterior superior margin of the anterior iliac crest to preserve the lateral femoral cutaneous nerve. After the dissection of the skin and the underlying soft tissues, the superior surface of the iliac crest was exposed. Then the GMED, GMAX, and ILIAC muscle attachments covering the medial and lateral surfaces of the iliac crest were dissected subperiosteally to expose the bone surfaces. After the completion of the dissection process, a tricortical autogenous bone block was harvested by using a microsaw and a chisel osteotomy. Then, the sharp and rough contours were smoothed, and mini-wac drains were placed to the donor site to control postoperative edema. Finally, the three-layered closure including the periosteum, muscles, and skin was performed to obtain primer closure of the wound. 1/0 resorbable polyglactin 910 sutures (Vicryl, Ethicon, Somerville, NJ, USA) were used for the closure of the periosteum. Muscle attachments and subcutaneous soft tissue layers were closed by using 3/0 resorbable polyglactin 910 sutures (Vicryl, Ethicon, Somerville, NJ, USA), and the skin incision was sutured with 3/0 nonresorbable polypropylene sutures (Prolen; Dogsan Medical Supplies Industry, Trabzon, Turkey). Patients were hospitalized for 1 day to control early postoperative complications, and postsurgical medications including antibiotics, analgesics, and corticosteroids were prescribed. The patients were administered antibiotics for 7 days starting on the day of the operation (1,000 mg of amoxicillin and clavulanic acid twice daily or 600 mg of clindamycin for the patients who have penicillin allergy twice a day) and analgesics (600 mg ibuprofen every 6 hours for the first day and, if needed, for other days). As a corticosteroid, dexamethasone (8 mg daily) was administered for 2 days to control the postoperative edema. A day after the surgery, mini-wac drains were removed and the patients were discharged with detailed written postoperative instructions. Nonresorbable sutures used for the skin closure were removed 10 to 12 days after the surgery and the healing period was uneventful.

### 2.3. Gait Experiments

Temporospatial and kinematic (lower limb joint angles) gait data were collected from the patients at the Istanbul University, Faculty of Medicine, Motion Analysis Laboratory. Each patient was asked to walk as a natural way at a self-selected speed during pre- and postoperative periods in the laboratory. Three postoperative periods were specified as one week (post-op1), two weeks (post-op2), and three weeks (post-op3) after the surgery.

Reflective passive markers were mounted on the specific anatomic regions of the patients as described by Davis et al. [21], and three-dimensional position data of the markers were recorded by using six optical cameras (ELITE2002; BTS, Milan, Italy) of which sampling rate was 100 Hz. A second-order Butterworth low-pass filter (6 Hz) was applied to smooth the marker trajectories. Joint angles were calculated from the marker data by means of the inverse kinematic technique. The ground reaction force was also measured simultaneously using two force plates (Kistler, Switzerland). Three gait trials were collected for each patient and averages of the temporospatial, kinematic, and muscle force data were calculated.

### 2.4. Muscle Force Calculation

Forces of the GMED, GMAX, and ILIAC muscles were calculated by using OpenSim, a musculoskeletal modeling and simulation program allowing the calculation of the human muscle forces using inverse dynamics and forward dynamics methods [22]. The human musculoskeletal model, which is available in OpenSim library (Gait2354 model), was used in the gait simulations. The model had 23 degree-of-freedom, 10 body segments, and 54 muscle-tendon actuators. To scale the inertial properties and dimensions of the generic musculoskeletal models according to the anthropometric properties of each patient, the scaling procedure was performed in OpenSim (version 3.3). Dimensions of each segment of each patient's model were scaled such that the distances between the virtual markers, which are placed on the unscaled musculoskeletal model, matched the distances between the experimental markers.

Static optimization (SO) was implemented for the calculation of individual muscle forces. In SO, a cost function, which is subjected to some physiologically based constraints, is optimized independently for each time point of interest [23, 24]. In the present study, SO was implemented by minimizing the sum of the squares of all muscle activations subject to the force-length and force-velocity properties of the muscles at each instant of the gait cycle [25].

### 2.5. Data Analysis

Since the grafting operation took place in the right iliac crest, all temporospatial, kinematic, and muscle force values were analyzed only for the right side of the body. To be able to quantitatively assess the recovery process of the patients, temporospatial, kinematic, and muscle force values were analyzed using different metrics and statistical methods. For temporospatial parameters, mean and standard deviation of the data were calculated. Joint angles were examined using the mean and standard deviation of the peak value and range of motion (RoM) of the corresponding joint. Muscle forces of the GMED, GMAX, and ILIAC calculated from SO were evaluated using the root mean square difference (RMSD) and the Pearson correlation coefficient (PCC), which were calculated between the pre- and postoperative muscle forces. If the value of RMSD is 0.01, it implies a mean error between pre- and postoperative muscle force of 1%. PCC is a measure of the resemblance between two curves, and if PCC value between two curves is 1, it means that pre- and postoperative muscle forces show a perfect agreement.

Statistical significance analysis was carried out by using SPSS software (Version 21.0; SPSS; Chicago, IL, USA). The level of significance was set at 0.05. Shapiro-Wilk test was performed to test the normalization of the data. All parameters were statistically analyzed using the one-way repeated-measure ANOVA. Bonferroni post hoc test was implemented to determine the significant difference between paired groups, if any. The differences between paired groups were evaluated at a level of significance of 0.012 (*p* < 0.012).

## 3. Results

Mean (±standard deviation) values of the pre- and postoperative temporospatial gait parameters are given in Table 1. It can be deduced from the table that stance time, step length, stride length, and mean velocity significantly decreased during post-op1 and continued to increase during post-op1 and post-op2 when compared to pre-op parameters. All temporospatial parameters, except double support time, measured in post-op3 are closer to those in pre-op than those in post-op1 and post-op2.

Mean (±standard deviation) values of the kinematic gait parameters for the pelvis and hip, knee, and ankle joints are given in Table 2. As similar as in the temporospatial parameters, kinematic parameters measured in post-op3 were closer to those measured in pre-op than those in post-op1 and post-op2. All kinematic parameters, except RoM of pelvic tilt, mean pelvic tilt, mean hip abduction and adduction, mean hip extension, and peak knee extension, reduced in post-op1 when compared to pre-op parameters and increased while the recovery period remains. The RoM of pelvic tilt increased from 4.07° to 5.29° during post-op1 and decreased to 3.67° at the end of post-op3. The RoM of hip flexion dropped from 40.04° to 11.8° in post-op1 and increased to 39.82° while the recovery duration remains. RoM of knee flexion decreased from 53.40° to 26.48° in post-op1, and it began to increase in post-op2 and reached to 54.69° in post-op3. The same pattern can be observed in peak knee flexion as well. Nevertheless, peak knee flexion at initial contact and midstance increased during post-op1 and reduced in post-op3. RoM of ankle dorsiflexion dropped from 25.91° to 9.49° in post-op1 and then increased to 24.64° in post-op3. While peak ankle dorsiflexion showed a similar trend with RoM of ankle dorsiflexion, peak ankle plantar flexion increased in post-op1 and reduced in post-op3.

Four kinematic gait parameters were taken into account to determine if patients had a stiff knee gait pattern [26]. These parameters are (*i*) peak knee flexion angle, (*ii*) range of knee flexion in early swing measured from toe-off to peak flexion, (*iii*) total range of knee motion, and (*iv*) timing of peak knee flexion in swing. If the value was more than two standard deviations below the average control value from healthy subjects in the case of parameters *i*-*iii*, or more than two standard deviations above the average control value in the case of parameter *iv*, it can be indicative of stiff knee gait. A patient is considered to show stiff knee characteristics if three or more of these parameters were indicative of stiff knee gait [26, 27]. In our case, all the patients met the inclusion criteria for post-op1, and their gait characteristics can be classified as the stiff gait for the first week after surgery (Table 3).

**Figure 1:**
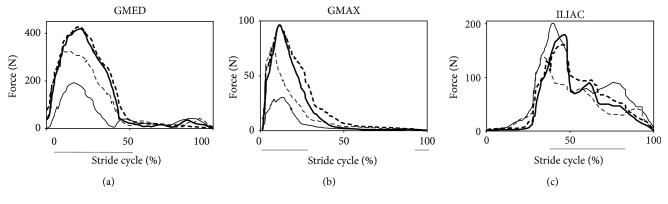
Comparison of the muscle forces computed from static optimization for pre- and postoperative periods (a) GMED, (b) GMAX, and (c) ILIAC muscles. The horizontal solid bars indicate the periods of experimental EMG activity obtained from the literature [28, 29]. Solid bold line: pre-op; solid thin line: post-op1; thin dashed line: post-op2; bold dashed line: post-op3.

Muscle force changes of the GMED, GMAX, and ILIAC over one stride are given in Figure 1. To validate the accuracy of the sequence and timing of the calculated muscle forces, experimental electromyography (EMG) recorded during gait tasks from healthy subjects and reported in the literature was used [28, 29]. It was observed that the timings of the muscle force simulations and experimental EMG data were in good agreement (Figure 1). According to muscle force prediction results, postoperative muscle forces approached to the preoperative characteristics while the recovery process was progressing. Forces of all three muscles calculated during post-op3 showed the best match to the preoperative muscle forces among the three postoperative periods.

**Figure 2:**
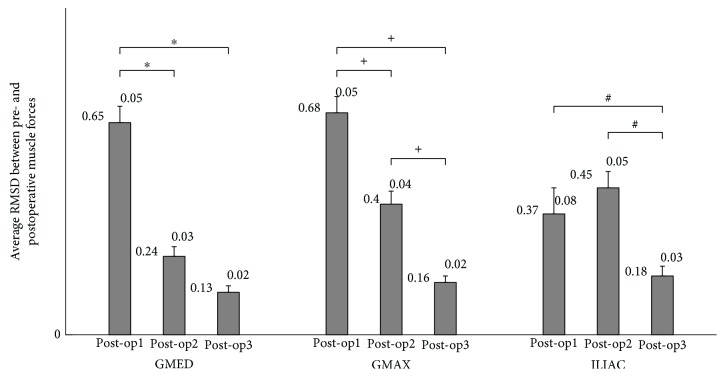
Mean (±standard deviation) root mean square difference (RMSD) values between the pre- and three postoperative muscle forces. ^∗^, +, # denote the statistical significance among intragroup comparisons.

**Figure 3:**
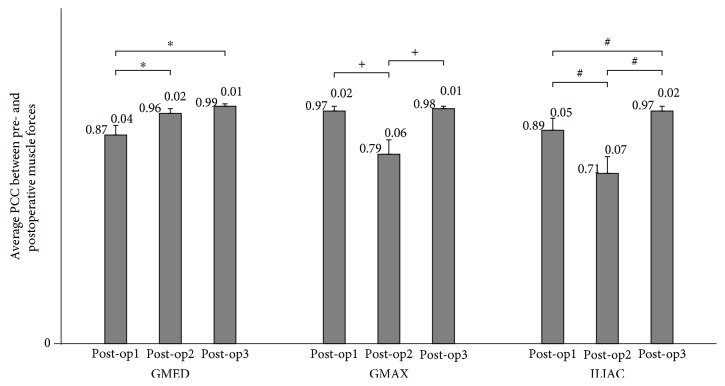
Mean (±standard deviation) Pearson correlation coefficient (PCC) values between the pre- and three postoperative muscle forces. ^∗^, +, # denote the statistical significance among intragroup comparisons.

The average RMSD and PCC values calculated between the pre- and postoperative muscle forces are given in Figures 2 and 3, respectively. Muscle forces calculated in post-op3 showed the best agreement with those calculated in pre-op than post-op1 and post-2 in terms of both magnitude (Figure 2) and correlation (Figure 3).

## 4. Discussion

Various types of bone grafts have been used for the reconstruction of bone defects for more than a century, and anterior iliac crest bone grafting is considered as the best option because of its functional and structural superiorities [30, 31]. Although anterior iliac crest bone grafting is considered a safe and relatively easy operation, complications of this surgical technique have been reported by many researchers [6, 13, 32, 33]. In addition to general surgical adversities, fracture of the ilium and damage to the acetabular fossa and surrounding muscle attachments may lead to specific complications [34]. Most of the surgeons believe that reduced soft tissue trauma and avoidance of intraoperative complications would diminish donor site morbidity and gait disturbance [34–36]. General complications and morbidity associated with anterior iliac crest bone grafting are well-documented [34] but the gait disturbance is less certain. Sudhakar et al. [19] stated that early recovery of gait disturbance is directly related with the protection of the neighboring muscles, iliac spine, and tensor fascia lata from trauma. In addition to a traumatic surgery, effective pain management is highly related to gait disturbance. Although some studies do not confirm, it is generally accepted that the average recovery period for gait disturbance after iliac crest bone grafting varies between two and four weeks [17–19, 36].

In this study, we aimed to evaluate the gait functions of patients, who underwent iliac crest bone grafting in atrophic jaw augmentation operation, in the early recovery course. By comparing the pre- and postoperative gait characteristics of the patients, we found that there were significant differences in the temporospatial and kinematic gait parameters and muscle forces between the pre- and postoperative periods, especially between pre-op and post-op1 periods. We observed that remarkable progress was made in the improvement of the locomotor function from the second week; however, patients were able to reach their normal walking patterns from the third week.

In this study, although the stance time of one gait cycle reduced significantly from 58% to 53% from pre-op to post-op1, which may be attributed to the antalgic gait, no significant reduction was seen between pre-op and post-op2 and post-op3 (59%±1.3, 59%±0.9, respectively). RoM of the hip, knee, and ankle joints in the sagittal plane obtained for pre-op, post-op2, and post-op3 are so close to each other. Both these findings implied that preoperative gait kinematics was recovered from the second week. On the other hand, we have found that the forces of all three muscles calculated during post-op3 showed the best match to the preoperative muscle forces among the three postoperative periods (there was a statistical difference between the muscle forces calculated for post-op2 and post-op3), indicating that full muscle recovery did not start from the second week after surgery but from the third week (Figures 2 and 3). Furthermore, we found a statistical difference between the mean pelvic tilt angles and RoMs of pelvic rotation for post-op2 and post-op3 periods, which also pointed out that pelvis kinematics returned to its preoperative characteristics from the third week of the surgery.

We have noticed that patients showed stiff knee gait characteristics within the first week after surgery but not in the second and third weeks following the surgery (Table 3). However, the observed stiff knee pattern may be the result of reduced mean gait velocity which dropped from 0.87 m/s to 0.60 m/s from pre-op to post-op1. Also, we qualitatively observed antalgic gait in the patients during the post-op1 gait tasks, which may be attributed to the occurrence of the stiff knee gait patterns in post-op1 [37, 38].

There are a number of limitations that should be considered when interpreting the results. First, muscle forces taken into account in the study were predictions that are not experimentally obtained actual forces. Due to the ethical concerns, it is nearly impossible to obtain directly measured forces from intact muscles of the human body [39]. Computational approaches such as OpenSim or AnyBody seem to be the most practical and acceptable alternatives to calculate muscle forces [22, 40]. Second, although we implemented the scaling procedure to account for the different anthropometry of the patients in the musculoskeletal models, the scaling may not precisely reflect all of the anatomical and morphological differences present. EMG is commonly used to test the validity of model-predicted muscle forces in terms of the sequence and timing of muscle activity [41]. Since the ILIAC muscle is located in the deepest region of the trunk, the electrical activity of the ILIAC seems unmeasurable with surface EMG [42]. Furthermore, since the patients were reluctant to enable us to record EMG signals from the GMAX and GMED muscles due to privacy, we could not record EMG signals in our study, which can be considered as another limitation. Therefore, predicted muscle forces should be interpreted with caution.

## 5. Conclusions

In conclusion, most of the temporospatial and kinematic gait parameters and all the muscle forces of post-op3 were closer to pre-op patterns than those of post-op1 and post-op2. The patients began to regain their preoperative gait characteristics from the second week after surgery, but complete recovery in gait was observed three weeks after the surgery. We expect that the findings of this study concerning the alterations in the gait parameters and muscle force production capabilities would contribute to the determination of the rehabilitation programs for the patients with locomotion deficits following anterior iliac bone grafting.

## Figures and Tables

**Table 1:** Mean (±standard deviation) values of temporospatial gait parameters obtained during pre-op, post-op1, post-op2, and post-op3 periods.

Temporospatial parameters	Pre-op	Post-op1	Post-op2	Post-op3	Statistical significance
Stance time (ms)	790 ± 10	567 ± 25	760 ± 21	790 ± 14	*p-p*_1_; *p*_1_*-p*_2_ *p*_1_*-p*_3_
Stance time (% gait cycle)	58 ± 0.6	53 ± 1.6	59 ± 1.3	59 ± 0.9	*p-p*_1_; *p*_1_*-p*_2_ *p*_1_*-p*_3_
Cadence (step/min)	89 ± 2.5	113 ± 4.7	94 ± 3.8	89 ± 2.9	*p-p*_1_; *p*_1_*-p*_2_ *p*_1_*-p*_3_
Double support time (ms)	120 ± 10	150 ± 15	140 ± 13	160 ± 11	*p-p*_1_; *p-p*_3_
Double support (% gait cycle)	9 ± 1	14 ± 1.2	11 ± 1.1	12 ± 0.5	*p-p*_1_
Step length (mm)	536 ± 40	349 ± 46	612 ± 45	534 ± 43	*p-p*_1_; *p*_1_*-p*_2_ *p*_1_*-p*_3_
Stride length (mm)	1169 ± 15	651 ± 55	1217 ± 40	1082 ± 20	*p-p*_1_; *p*_1_*-p*_2_ *p*_1_*-p*_3_; *p-p*_2_ *p*_2_*-p*_3_
Step width (mm)	141 ± 3	211 ± 12	156 ± 9	139 ± 5	*p-p*_1_; *p*_1_*-p*_2_ *p*_1_*-p*_3_
Mean velocity (m/s)	0.87 ± 0.4	0.60 ± 0.24	0.96 ± 0.4	0.82 ± 0.5	*p-p*_1_; *p*_1_*-p*_2_ *p*_1_*-p*_3_

*p-p*_1_: statistical significance between pre-op and post-op1. *p-p*_2_: statistical significance between pre-op and post-op2. *p-p*_3_: statistical significance between pre-op and post-op3. *p*_1_*-p*_2_: statistical significance between post-op1 and post-op2. *p*_1_*-p*_3_: statistical significance between post-op1 and post-op3. *p*_2_*-p*_3_: statistical significance between post-op2 and post-op3.

**Table 2:** Mean (±standard deviation) values of kinematic gait parameters obtained during pre-op, post-op1, post-op2, and post-op3 periods.

Kinematics parameters	Pre-op (Deg)	Post-op1 (Deg)	Post-op2 (Deg)	Post-op3 (Deg)	Statistical significance
*Pelvis*					
RoM pelvic obliquity	4.67 ± 0.2	4.13 ± 0.3	5.03 ± 0.4	4.03 ± 0.2	*p-p*_1_; *p-p*_3_ *p*_1_*-p*_2_; *p*_2_*-p*_3_
RoM pelvic tilt	4.07 ± 0.1	5.29 ± 0.1	4.13 ± 0.1	3.67 ± 0.3	*p-p*_1_; *p*_1_*-p*_2_*;p*_1_*-p*_3_
Mean pelvic tilt	10.27 ± 0.3	12.05 ± 0.3	13.10 ± 0.1	10.20 ± 0.3	*p-p*_2_; *p*_1_*-p*_3_*;p*_2_*-p*_3_
RoM pelvic rotation	12.16 ± 0.2	6.92 ± 0.3	7.89 ± 0.1	12.51 ± 0.3	*p-p*_1_; *p-p*_2_ *p*_1_*-p*_3_; *p*_2_*-p*_3_
*Hip*					
Mean hip abd/add	-4.47 ± 0.1	-13.15 ± 0.1	-5.57 ± 0.2	-6.63 ± 0.1	*p-p*_1_; *p*_1_*-p*_2_; *p*_1_*-p*_3_
Peak hip ext	-4.95 ± 0.3	21.46 ± 0.2	-5.78 ± 0.1	-9.04 ± 0.2	*p-p*_1_; *p*_1_*-p*_2_ *p*_1_*-p*_3_
Peak hip flex	35.08 ± 0.3	33.26 ± 0.2	33.29 ± 0.3	30.78 ± 0.1	*p-p*_3_
RoM hip flex/ext	40.04 ± 0.2	11.8 ± 0.2	39.07 ± 0.3	39.82 ± 0.1	*p-p*_1_; *p*_1_*-p*_2_; *p*_1_*-p*_3_
RoM hip rotation	11.01 ± 0.1	9.42 ± 0.1	13.22 ± 0.5	9.97 ± 0.2	*p-p*_1_; *p-p*_2_ *p*_1_*-p*_2_; *p*_2_*-p*_3_
*Knee*					
RoM knee flex/ext	53.40 ± 0.4	26.48 ± 0.3	52.06 ± 0.3	54.69 ± 0.4	*p-p*_1_; *p*_1_*-p*_2_; *p*_1_*-p*_3_
Peak knee flex/ext at initial contact	12.36 ± 0.2	18.77 ± 0.1	10.72 ± 0.2	6.58 ± 0.4	*p-p*_1_; *p-p*_3_ *p*_1_*-p*_3_; *p*_1_*-p*_2_
Peak knee ext at midstance	7.49 ± 0.1	21.69 ± 0.2	2.89 ± 0.1	2.95 ± 0.1	*p-p*_1_; *p-p*_2_ *p-p*_3_; *p*_1_*-p*_2_; *p*_1_*-p*_3_
Peak knee flex	60.89 ± 0.3	48.17 ± 0.4	54.95 ± 0.4	57.64 ± 0.3	*p-p*_1_; *p*_1_*-p*_3_
*Ankle*					
RoM ankle dorsi/plantar flex	25.91 ± 0.3	9.49 ± 0.1	22.13 ± 0.4	24.64 ± 0.3	*p-p*_1_; *p*_1_*-p*_2_; *p*_1_*-p*_3_
Peak ankle dorsi flex	10.89 ± 0.2	10.06 ± 0.3	10.90 ± 0.2	13.47 ± 0.2	*p-p*_3_; *p*_1_*-p*_3_; *p*_2_*-p*_3_
Peak ankle plantar flex	-15.01 ± 0.2	0.57 ± 0.1	-11.24 ± 0.2	-11.18 ± 0.2	*p-p*_1_; *p*_1_*-p*_2_; *p*_1_*-p*_3_

*p-p*_1_: statistical significance between pre-op and post-op1. *p-p*_2_: statistical significance between pre-op and post-op2. *p-p*_3_: statistical significance between pre-op and post-op3. *p*_1_*-p*_2_: statistical significance between post-op1 and post-op2. *p*_1_*-p*_3_: statistical significance between post-op1 and post-op3. *p*_2_*-p*_3_: statistical significance between post-op2 and post-op3. RoM: range of motion; abd: abduction; add: adduction; ext: extension; flex: flexion.

**Table 3:** Four gait parameters as measures of whether a patient had a stiff knee gait pattern.

	Pre-op	Post-op1	Post-op2	Post-op3
Peak knee flexion angle (Deg)	60.89	48.17	54.95	57.64
Range of knee flexion in early swing measured from toe-off to peak flexion (Deg)	28.91	7.33	31.57	38.52
Total range of knee motion (Deg)	53.4	26.48	52.06	54.69
Timing of peak knee flexion in swing	% 12	% 9	% 15	% 13

Standard deviations are 4.1, 5.23, 3.8, and 2.10 for (*i*) peak knee flexion angle, (*ii*) range of knee flexion in early swing measured from toe-off to peak flexion, (*iii*) total range of knee motion, and (*iv*) timing of peak knee flexion in swing, respectively.

## Data Availability

The gait data used to support the findings of this study are available from the corresponding author upon request.
